# Supplementary material for: DVT-primed neutrophils reshape the brain microenvironment to promote brain metastasis
Source: bioRxiv. 2026 Sep 17:2026.09.15.751589. Preprint. [Version 1] doi: 10.64898/2026.09.15.751589 (PMC13596369; doi:10.64898/2026.09.15.751589)
Supplement: Supplementary file 1 [file NIHPP2026.09.15.751589v1-supplement-1.pdf]

bioRxiv preprint doi: https://doi.org/10.64898/2026.09.15.751589; this version posted September 17, 2026. The copyright holder for this preprint (which was not certified by peer review) is the author/funder. This article is a US Government work. It is not subject to copyright under 17 USC 105 and is also made available for use under a CC0 license.

Supplementary Figure 1.

(A) Schematics of TriNetX data analysis. (B-C) Representative images of thrombus at endpoint (Day 12), E0771-BR5 (B), 4T1-BR5 (C). (D) Representative image of immunofluorescent staining of MPO and citrullinated histone (cHis) in thrombi at endpoint (Day 12) (4T1-BR5). (E-H) Spearman correlation between brain metastasis area and thrombus length and weights (E-F: E0771-BR5, G-H: 4T1-BR5) (I) Schematic of mammary fat pad injection and spontaneous lung metastasis model (4T1) (J) Tumor growth curve of mammary fat pad injection of 4T1 (n = 9 for sham, n = 8 for DVT). (K) Quantification of lug metastasis area (Day 21, (n = 9 for sham, n = 8 for DVT). (L) Quantification of the number of lug metastasis lesions (Day 21, (n = 9 for sham, n = 8 for DVT).

Supplementary Figure 2.

(A) Dynamics of neutrophil count in peripheral blood (E0771-BR5). (B) Dynamics of neutrophil count in peripheral blood (4T1-BR5). (C-E) Flow cytometric analysis of the proportions of CD3+ T cells (C), CD4+ T cells (D), and CD8+ T cells (E) in peripheral blood (E0771-BR5R). (F) Gating strategy of peripheral blood for neutrophils in the neutrophil depletion animal model (E0771-BR5). (G) Dynamics of neutrophil count in peripheral blood in the neutrophil depletion animal model (E0771-BR5). (H) Schematics of the DVT model with i.c. injection of eGFP-labelled E0771-BR5 cells. (I) Quantification of the number of

bioRxiv preprint doi: https://doi.org/10.64898/2026.09.15.751589; this version posted September 17, 2026. The copyright holder for this preprint (which was not certified by peer review) is the author/funder. This article is a US Government work. It is not subject to copyright under 17 USC 105 and is also made available for use under a CC0 license.

extravasated eGFP-positive E0771-BR5 cancer cells. (J) Quantification of Ki-67 positive cancer cells among the eGFP-positive E0771-BR5 cancer cells.

Supplementary Figure 3.

(A) Neutrophil surface markers of peripheral blood collected from 4T1-BR5 injected mice (Day5). (B) Neutrophil surface markers of peripheral blood collected from tumor naïve C57/B6 mice (2 days post surgery) (C) Neutrophil surface markers of peripheral blood collected from tumor naïve BALC/c mice (2 days post surgery).

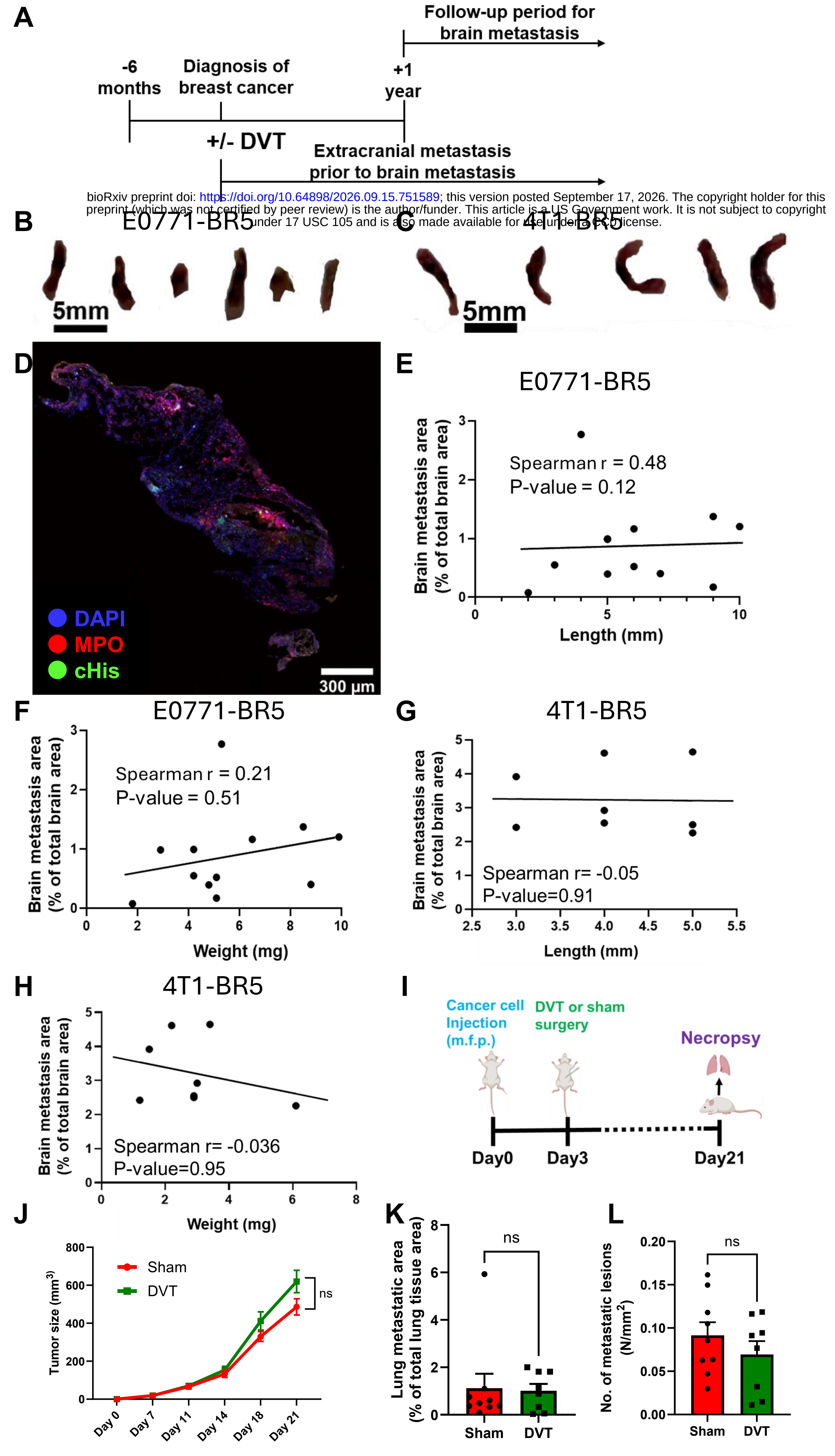

bioRxiv preprint doi: https://doi.org/10.64898/2026.09.15.751589; this version posted September 17, 2026. The copyright holder for this preprint (which was not certified by peer review) is the author/funder. This article is a US Government work. It is not subject to copyright under 17 USC 105 and is also made available for use under a CC0 license.

Supplementary Figure 1.

bioRxiv preprint doi: https://doi.org/10.64898/2026.09.15.751589; this version posted September 17, 2026. The copyright holder for this preprint (which was not certified by peer review) is the author/funder. This article is a US Government work. It is not subject to copyright under 17 USC 105 and is also made available for use under a CC0 license.

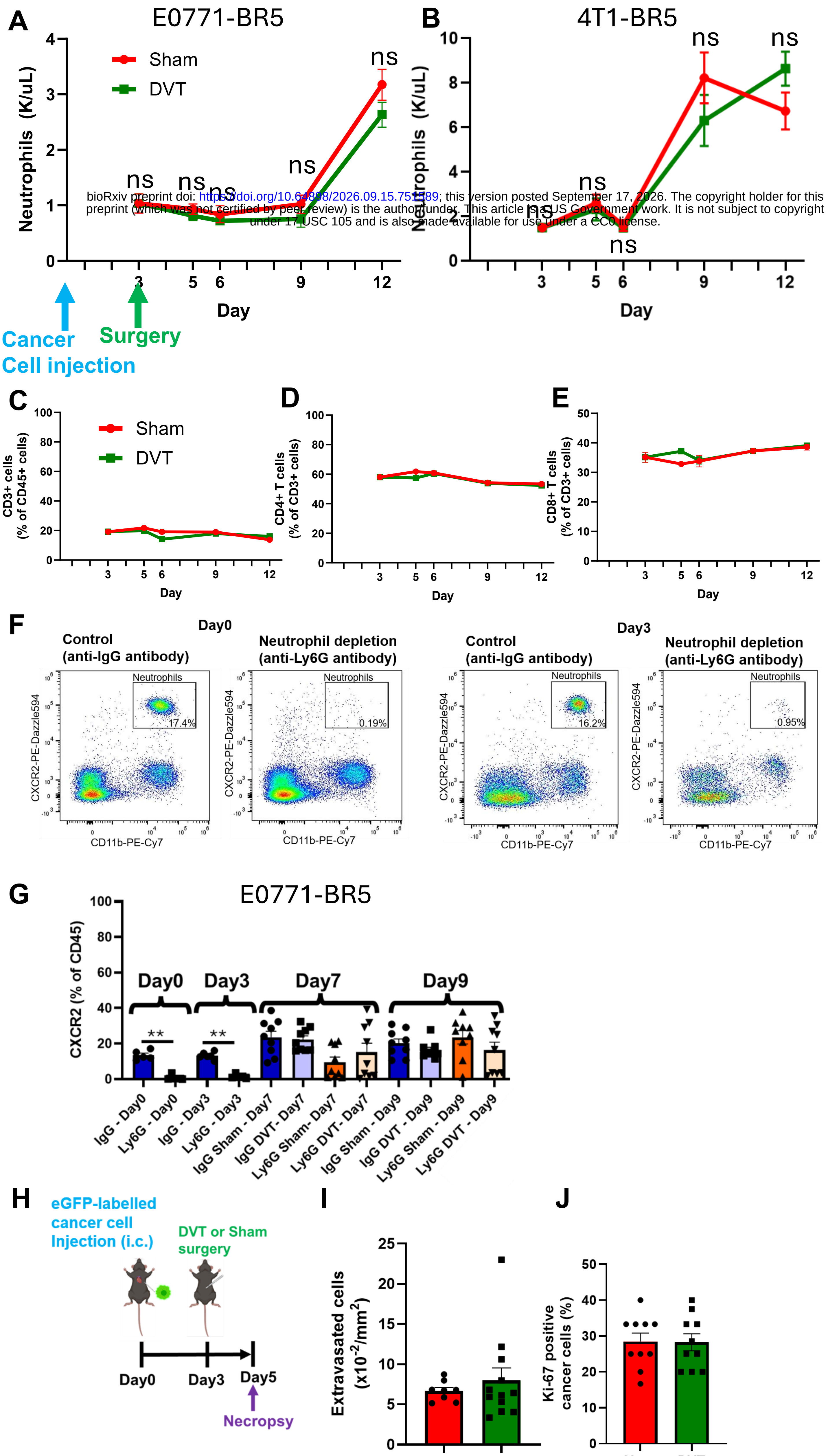

Supplementary Figure 2.

bioRxiv preprint doi: https://doi.org/10.64898/2026.09.15.751589; this version posted September 17, 2026. The copyright holder for this preprint (which was not certified by peer review) is the author/funder. This article is a US Government work. It is not subject to copyright under 17 USC 105 and is also made available for use under a CC0 license.

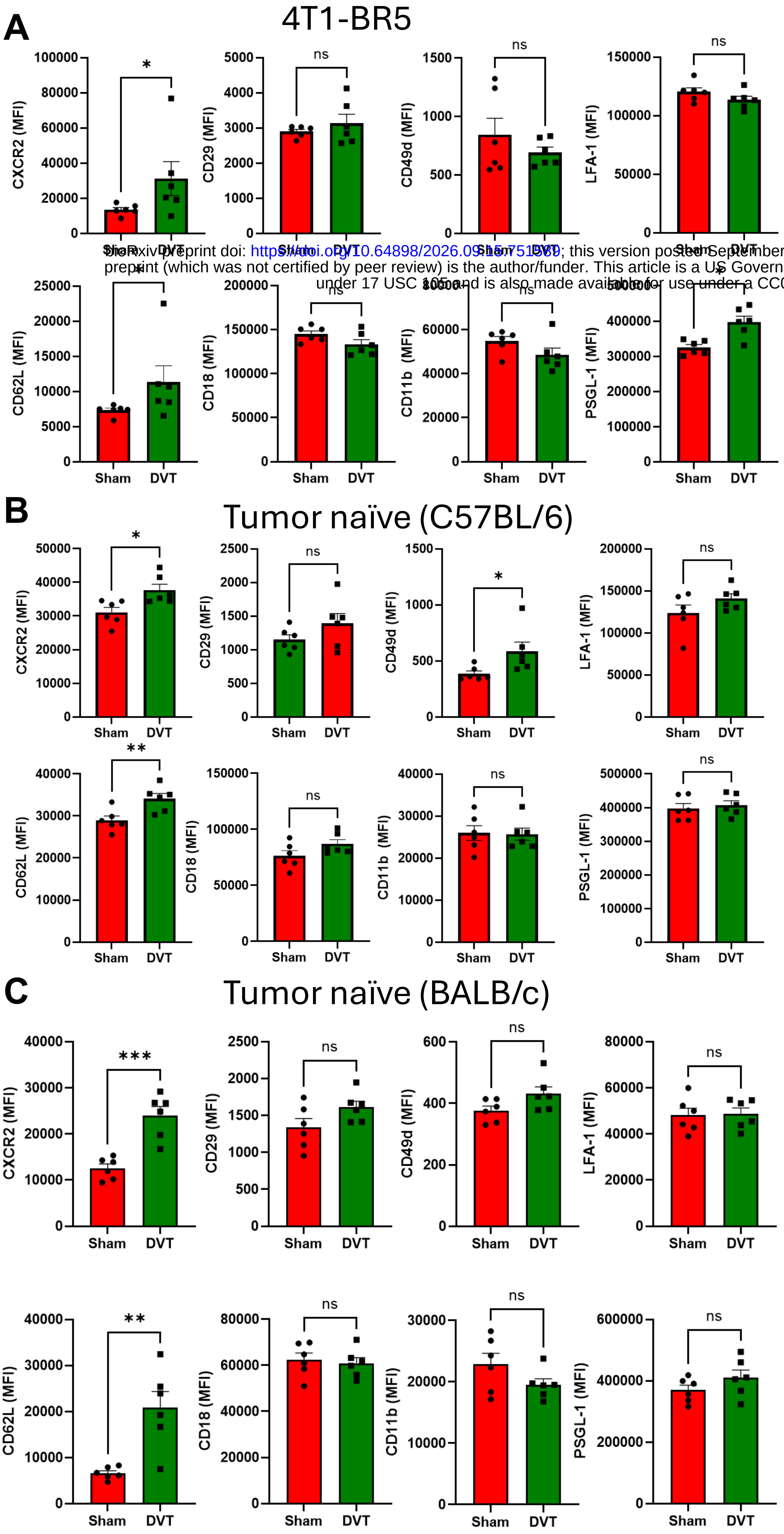

Supplementary Figure 3.

Supplementary Table 1.

		No DVT (N=2,511)	DVT (N=2,511)
**Age at diagnosis**		65 (+/-13)	65 (+/-13)
**Race**			
	White	73.84%	72.16%
	Black or African American	11.15%	16.29%
	Asian	6.49%	3.74%
	Native Hawaiian or Other Pacific Islander	1.08%	0.68%
	American Indian or Alaska Native	0.40%	0.40%
	Unknown Race	5.34%	3.94%
	Other Race	1.95%	2.87%
**Ethnicity**			
	Not Hispanic or Latino	84.07%	82.36%
	Hispanic or Latino	2.55%	5.06%
	Unknown Ethnicity	13.38%	12.59%
**Estrogen receptor**			
	Positive	37.40%	37.32%
	Negative	14.66%	14.38%
**Progesterone receptor**			
	Positive	2.07%	2.39%
	Negative	1.24%	1.39%
**HER2 receptor**			
	Positive	1.04%	1.43%
	Negative	3.07%	3.39%
**Initial Stage**			
	Stage 0	0%	0.40%
	Stage 1	0.64%	0.60%
	Stage 2	0.40%	0.48%
	Stage 3	0.72%	1.00%
	Stage 4	2.79%	3.51%
**Extracranial metastasis sites**			
	Lymph nodes	26.25%	27.52%
	Bone	58.62%	56.39%
	Lung	21.74%	22.86%
	Liver	19.24%	19.91%
**Medical co-morbidities**			
	Cerebral infarction	3.54%	3.62%
	Hypertension	47.39%	47.59%
	Dyslipidemia	31.94%	32.42%
	Diabetes mellitus	20.35%	21.23%
	Chronic kidney disease	10.83%	11.11%
	Chronic ischemic heart disease	10.75%	11.91%
	Atrial fibrillation	6.69%	7.77%
	Acute myocardial infarction	2.67%	2.95%
	Pulmonary embolism	11.99%	12.39%
	Heart failure	8.68%	9.92%

Supplementary Table 2.

	No. of patients with extracranial metastasis	No. of patients who developed brain metastasis	Incidence of brain metastasis	Odds ratio	P-value
**No DVT**	2,326	65	2.79%	1.8	9.5×10^{-5}
**DVT**	2,226	112	5.03%		
